# Combined Use of Hyperbaric and Hypobaric Ropivacaine Significantly Improves Hemodynamic Characteristics in Spinal Anesthesia for Caesarean Section: A Prospective, Double-Blind, Randomized, Controlled Study

**DOI:** 10.1371/journal.pone.0125014

**Published:** 2015-05-13

**Authors:** ZheFeng Quan, Ming Tian, Ping Chi, Xin Li, HaiLi He, Chao Luo

**Affiliations:** Department of Anesthesiology, Beijing You An Hospital, Capital Medical University, Beijing, 100069, China; The University of Tokyo Hospital, JAPAN

## Abstract

**Purpose:**

To observe the hemodynamic changes of parturients in the combined use of hyperbaric (4 mg) and hypobaric (6 mg) ropivacaine during spinal anesthesia for caesarean section in this randomized double-blind study.

**Methods:**

Parturients (n = 136) undergoing elective cesarean delivery were randomly and equally allocated to receive either combined hyperbaric and hypobaric ropivacaine (Group A) or hyperbaric ropivacaine (Group B). Outcome measures were: hemodynamic characteristics, maximum height of sensory block, time to achieve T8 sensory blockade level, incidence of complications, Apgar scores at 1 and 5 min, and neonatal blood gas analysis.

**Results:**

Group A had a lower level of sensory blockade (T6 [T6-T7]) and longer time to achieve T8 sensory blockade level (8 ± 1.3 min) than did patients in Group B (T3 [T2-T4] and 5 ± 1.0 min, respectively; *P* < 0.001, both). The incidence rates for hypotension, nausea, and vomiting were significantly lower in Group A (13%, 10%, and 3%, respectively) than Group B (66%, 31%, and 13%; *P* < 0.001, *P* = 0.003, *P* = 0.028).

**Conclusions:**

Combined use of hyperbaric (4 mg) and hypobaric (6 mg) ropivacaine significantly decreased the incidences of hypotension and complications in spinal anesthesia for caesarean section by extending induction time and decreasing the level of sensory blockade.

**Trial Registration:**

Chinese Clinical Trial Register ChiCTR-TRC-13004622

## Introduction

Hyperbaric anesthetics are used more often than hypobaric or isobaric for spinal anesthesia during caesarean section, due to their better anesthetic effects and controllability [1–8]. However, after use of hyperbaric anesthetics the incidence of hypotension is as much as 60–90% during the perioperative period [9–15]. This is related to both the rapid onset of analgesia in spinal anesthesia and the characteristics of hyperbaric anesthetics, such as the uncontrolled upper plane of anesthesia [16,17].

We hypothesized that the unwanted effect of hypotension associated with use of hyperbaric anesthetics for spinal anesthesia during caesarean section may be ameliorated by utilizing the properties of both hyperbaric and hypobaric solutions, specifically by administering 4 mg of hyperbaric anesthetic and 6 mg of hypobaric anesthetic successively into the intrathecal space through the L2-3 interspace. Theoretically, the spread of the first 4 mg of hyperbaric anesthetic would move in the cephalad direction (subsiding towards T6), and the 6 mg of hypobaric anesthetic would be inclined to spread caudally (floating towards L3). This unique technique could avoid the spinal characteristic moving to one side and easily control the upper plane of anesthesia by adjusting the dose of hypobaric anesthetics.

In the present study, we assessed the hemodynamic consequences associated with the combined use of hyperbaric and hypobaric ropivacaine during spinal anesthesia for caesarean section.

## Materials and Methods

The supporting CONSORT checklist is available as supporting information; see S1 CONSORT Checklist and S1 Protocol (S1 Protocol Translation).

### Ethics statement

This prospective, double-blind, randomized, controlled study was approved by the Ethics Committee of Beijing You An Hospital in China (IRB No. 2013–11), and is registered in the Chinese Clinical Trial Register (ChiCTR-TRC-13004622). This study was registered prior to enrolling subjects in the study. All patients provided written informed consent prior to inclusion.

### Study design and subject allocation

This single-center study was designed in 2013 and performed at Beijing You An Hospital, a teaching hospital of Capital Medical University (China). Included in this study were 140 patients underwent elective cesarean section between may 2014 and August 2014. ASA physical status I or II (gestational age ≥37 weeks, singleton), undergoing elective cesarean delivery under combined spinal-epidural anesthesia. The exclusion criteria were: age <18 years; height <150 cm or >180 cm; weight <50 kg or >100 kg; hypertension; multiple pregnancy; placenta previa; cardiovascular or cerebrovascular diseases; known abnormal fetal development; contraindications for intraspinal anesthesia; or signs of labor onset. Hypertensive patients were excluded from this study, even if controlled with medication.

All patients underwent preoperative fasting for 8 hours and water deprivation for more than 4 hours. The patients were placed in a supine position with a 15° left lateral tilt after entering the operating room. The patient was administered 7 mL/kg of lactated Ringer's injection, within 10 min, via a 16-gauge cannula placed in a forearm vein. The infusion speed was then adjusted to 7 mL·kg^-1^·h^-1^. Blood pressure was monitored 5 min after infusion, and the mean value of three consecutive detections was recorded.

Patients were randomly allocated into two groups (Group A: Combined use of hyperbaric and hypobaric ropivacaine; Group B: Controls) using a computer-generated table. The investigator responsible for the random allocation and preparation of spinal anesthetics was not present during surgery or postoperative evaluations. This study was double-blinded, and we deduce that all the patients were blinded due to the nature of the study. The anesthetists responsible for outcome evaluations were blinded to the group assignment.

The anesthetics used in both groups consisted of two parts. Group A received from syringe A1, 0.8 mL of 0.5% hyperbaric ropivacaine, containing 4 μg fentanyl and 4% glucose; and from syringe A2, 1.2 mL of 0.5% hypobaric ropivacaine, containing 6 μg fentanyl and sterile distilled water. Group B, the control, received from syringe B1, 0.8 mL of 0.5% hyperbaric ropivacaine, containing 4 μg fentanyl and 4% glucose; and from syringe B2, 1.2 mL of 0.5% hyperbaric ropivacaine, containing 6 μg fentanyl and 4% glucose.

A combined spinal-epidural procedure was performed at the L2-3 interspace with the patient maintained in the right lateral decubitus position. In each group of patients, the anesthetics in syringes 1 and 2 were injected successively when the needle entered the subarachnoid space. Thus, hyperbaric and hypobaric ropivacaine was successively injected and not injected after mixing. Injection speed in both groups was 0.1 mL/s. The spinal anesthesia needle was withdrawn and 3 cm of the epidural catheter was placed into the epidural space. Patients in whom insertion time of the epidural catheter was >3 min were excluded from the study. Then the investigator who was responsible for the random allocation announced the un-blinding of these patients. The upper sensory blockade level was checked by assessing the loss of pain sensation along the collarbone midline using a 20-gauge sterilized needle. The same investigator who was not present during other stages of this study applied this test. Blood pressure was determined at 1-min intervals until the beginning of the operation. If the T8 sensory blockade level was not achieved, an epidural supplement of 2% lidocaine was administered to maintain a T8 sensory level. All the anesthesia procedures were performed by a same anesthetist. Monitoring and observation of all the patients were conducted by another anesthetist. The same obstetrician performed all the caesarean sections. Patients who had an operative time >60 min were excluded from the study.

The perioperative period was monitored for the occurrence of hypotension. Hypotension was defined as systolic arterial pressure (AP) < 90 mmHg. When the perioperative systolic AP decreased to this level, 6 mg ephedrine was administered every 2 min until the systolic AP was maintained within normal range. Atropine (0.3 mg) was used to treat bradycardia (heart rate < 50 bpm).

The following were recorded: hemodynamic data, upper sensory blockade level, insertion time of the epidural catheter, complete analgesia time (beginning of intrathecal injection to a visual analogue scale [VAS] > 0), mean dose of ephedrine, time from the end of intrathecal injection to achievement of T8 sensory blockade level, operative time, volume of intraoperative blood loss, volume of intraoperative urine, and incidence of complications (nausea, vomiting, shivering, or dizziness). Neonatal conditions were assessed using Apgar scores at 1 and 5 min, and blood gas analyses of the umbilical artery and vein were performed.

### Statistical analyses

The primary outcome was the incidence of hypotension. The sample size was calculated according to our preliminary data. A minimum of 51 patients in each group would provide 90% statistical power to detect a 30% difference (from 50% to 20%) in the incidence of hypotension, at a 2-sided *P*-value < 0.05. Considering a presumed dropout rate of 20%, 70 patients were initially enrolled in each of the two groups.

SPSS 17.0 software was used for statistical analyses. Data were analyzed using the Shapiro-Wilk test to determine distribution. If normally distributed, the data are presented as mean ± standard deviation (SD) and two independent groups were compared using Student’s *t*-test. Data distributed non-normally are presented as median (min-max), and were analyzed using a Mann-Whitney U test. Categorical variables were analyzed using a chi-squared test, or Fisher’s exact test if the number of subjects in any contingency table cell was expected to be less than five. *P* < 0.05 was considered statistically significant.

## Results

**Fig 1:**
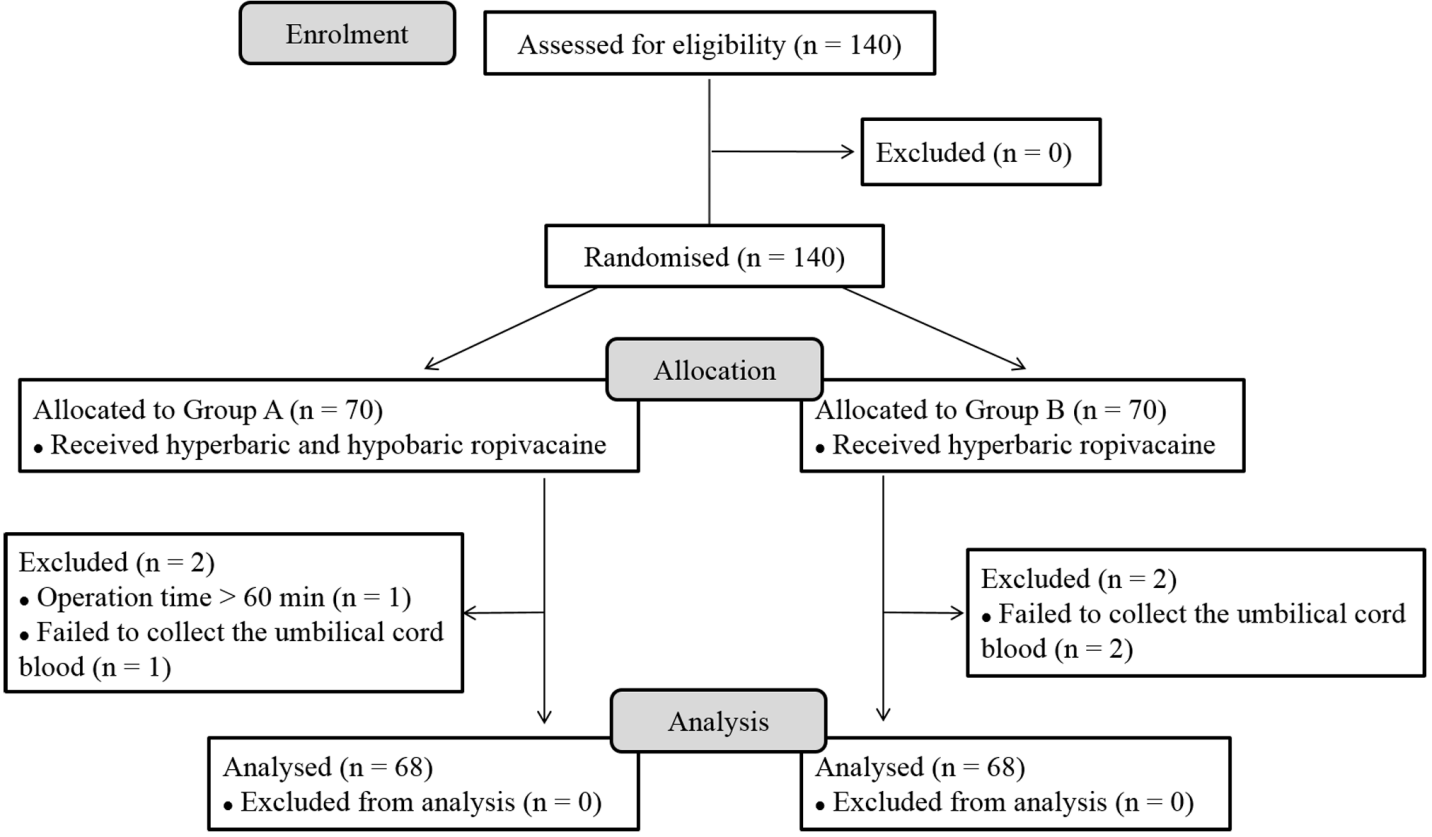
Study schematic for the patients undergoing elective caesarean section.

In total, 140 patients were assessed for study eligibility, and then randomized with 70 in each group. Four patients did not meet the inclusion criteria, among which one had an operative time >60 min (From Group A) and 3 failed to collect the umbilical cord blood (One from Group A and the other two from Group B). Finally, there were 68 patients analyzed in each group (Fig 1).

The baseline characteristics of the two groups were not significantly different, including insertion time of the epidural catheter, operative time, or volume of intraoperative transfusion, urine, or blood loss (Table 1). No supplemental anesthetics were administered into the epidural space or veins perioperatively in either group. No patients had a perioperative VAS > 0. Complete analgesia time was not statistically different between the groups (Table 1).

**Table 1:** Baseline characteristics of all patients during perioperative period and neonatal characteristics.*

			Group A	Group B	*P*
Patient	Age, y		27 (21–40)	28 (20–43)	0.277
	Height, cm		163 (152–173)	162 (154–175)	0.461
	Weight, kg		73 (52–91)	75 (58–88)	0.164
	Gestational age, wk		38 (37–41)	38 (37–40)	0.104
	Catheter insertion time, min		1.4 (1–2)	1.5 (1–2)	0.651
	Operative time, min		39 (26–45)	41 (29–54)	0.162
	Intraoperative transfusion, mL		868 ± 84	879 ± 69	0.414
	Intraoperative urine, mL		167 ± 52	176 ± 47	0.357
	Blood loss, mL		421 ± 57	415 ± 65	0.596
	Complete analgesia, min		139 ± 15	134 ± 17	0.119
	Baseline	Systolic AP, mmHg	133 ± 15	129 ± 12	0.119
		Diastolic AP, mmHg	62 ± 9	64 ± 10	0.174
		Mean AP, mmHg	85 ± 7	87 ± 7	0.283
		Heart rate, bpm	84 ± 10	81 ± 11	0.231
Neonate	Birth weight (g)		3344 ± 518	3250 ± 533	0.43
	Apgar at 1 min		9 (7–10)	9 (8–9)	0.89
	Apgar at 5 min		10 (8–10)	10 (9–10)	0.75
	Umbilical artery pH		7.29 ± 0.03	7.27 ± 0.05	0.01
	Umbilical vein pH		7.34 ± 0.05	7.35 ± 0.04	0.14

* Values presented as mean ± SD or median (min-max).

Neonatal umbilical artery blood pH was significantly higher in Group A (7.29 ± 0.03) than Group B (7.27 ± 0.05; *P* = 0.01). Other neonatal characteristics were comparable (Table 1).

Hemodynamic data, from intrathecal injection to delivery of the shoulder, suggested that the incidences of hypotension, systolic AP <100 mmHg, and systolic AP < 90 mmHg were all significantly lower in Group A (13%, 28%, and 10%, respectively) than Group B (66%, 90%, and 59%; P < 0.001, all; Table 2).

**Table 2:** Hemodynamic data and blockade characteristics of the patients.

	Group A	Group B	*P*
Incidence of hypotension, n (%)	9 (13%)	45 (66%)	<0.001
Systolic blood pressure <100 mmHg, n (%)	19 (28%)	61 (90%)	<0.001
Systolic blood pressure <90 mmHg, n (%)	7 (10%)	40 (59%)	<0.001
Mean dose of ephedrine, mg	11.5 ± 6.9	13.8 ± 8.5	0.287
Time to achieve T8 sensory blockade level, min	8 ± 1.3	5 ± 1.0	<0.001
Maximum height of sensory block	T6 (T6-T7)	T3 (T2-T4)	<0.001

The time required to achieve T8 sensory blockade level was longer in Group A (8 ± 1.3 min) than Group B (5 ± 1.0 min, *P* < 0.001). Group A also had a lower level of sensory blockade (T6 [T6-T7]) than patients in Group B (T3 [T2-T4]; *P* < 0.001; Table 2).

Group A experienced fewer incidences of nausea (10%) and vomiting (3%) than Group B (31% and 13%; *P* = 0.003 and *P* = 0.028, respectively). There were no statistical differences between the two groups in the incidence of other complications (Table 3).

**Table 3:** Incidence of patient complications. *

	Group A	Group B	*P*
Nausea	7 (10%)	21 (31%)	0.003
Vomiting	2 (3%)	9 (13%)	0.028
Shivering	41 (60%)	45 (66%)	0.475
Dizziness	0 (0%)	3 (4%)	0.244
Sleepiness	0 (0%)	0 (0%)	1.000

* Values presented as n (%)

## Discussion

Spinal anesthesia is the optimal choice for elective caesarean section. The advantages of spinal anesthesia include rapid onset and continuous spread of analgesia, and good analgesic effect. However, these properties cause perioperative hypotension in most patients [8,18–22]. In this study, Group A required more time to achieve a T8 sensory blockade level and reached a lower level of sensory blockade than patients in Group B. Therefore, Group A experienced a significantly lower rate of hypotension. In addition, the incidences of nausea and vomiting were significantly lower in Group A (10% and 3%, respectively) than Group B (31% and 13%; *P* = 0.003, *P* = 0.028). Neonatal umbilical artery blood pH was significantly higher in Group A (7.29 ± 0.03) than Group B (7.27 ± 0.05; *P* = 0.01). These data showed that the lower incidence of hypotension in patients was associated with fewer incidences of nausea and vomiting, and improved pH value of neonatal umbilical artery blood.

Our results showed that the anesthetic effect was similar between patients who received both hyperbaric and hypobaric solutions (Group A) and those who received the same dose of hyperbaric solution (Group B). There was no statistical difference in complete analgesia time between the two procedures, and either treatment could be used for caesarean sections performed within one hour. No supplemental anesthetic was administered into the epidural space or veins perioperatively in either group.

Currently, vasoactive agents and body position changes are considered the most effective methods to prevent or treat hypotension in spinal anesthesia during caesarean section [23–29]. However, vasoactive agents have the disadvantage of causing transient hypertension [15,30]. Thus, adjustment of body position may be the best method in caesarean section, and new progress has been mainly reported from studies on body position changes. Some studies have shown that the incidence of hypotension decreased when patients stayed seated for 5 min after the induction of spinal anesthesia before being turned to a supine position, because the sitting position decreased the upper sensory blockade level [18, 25]. Similar results were found in the present study.

In the current study, in Group A, hyperbaric and hypobaric solutions were administered successively into the intrathecal space through the L2-3 interspace. The hyperbaric solution spread towards T6, and the subsequent hypobaric solution rose to L3. Therefore, the sensory blockade level, especially the upper sensory blockade level, could be relatively controlled by adjusting the doses of solution with different densities. This combined procedure significantly prolonged induction time. This may be because, compared with single hyperbaric ropivacaine delivered in Group B, we used a decreased dose of hyperbaric ropivacaine in Group A. Thus, a lesser amount of spinal anesthetic spread towards T6 in the combined procedure. Theoretically, although the spread of spinal anesthetics towards T6 in Group B could be adjusted through body position changes, the actual effect is not satisfied. One reason may be that the physiological curve of the human lumbar segment could not be adjusted adequately by changes in body position. The isobaric solution can also prevent the spinal anesthetics moving to one side (T6) as in the combined procedure. However, the uncertainty of anesthesia effect limits the use of isobaric solution [31–36].

In this study, we chose a dose of 4 mg for hyperbaric ropivacaine in Group A. This is because our previous preliminary experiment showed that a smaller proportion of the hyperbaric ropivacaine in the combined procedure was associated with a longer induction time and more stable hemodynamics of the patient. Therefore, combined use of hyperbaric and hypobaric ropivacaine has the similar advantages of the single hyperbaric ropivacaine (compared to isobaric ropivacaine). In addition, this combined procedure could significantly reduce the higher level of anesthesia and the higher incidence of hypotension caused by single hyperbaric ropivacaine.

The preoperative preparation time of a surgeon is about 8 min (that is, from the end of spinal anesthesia to scalpel contact with the skin). Thus, to avoid any extra waiting time by the surgeon, we chose a suitable anesthetic combination (4 mg hyperbaric ropivacaine plus 6 mg hypobaric ropivacaine) in Group A to keep the induction time as close to 8 min as possible.

A limitation of this study is the post-randomization exclusions, which may undermine the effects of the random allocation of the intervention, and may ultimately introduce bias into the study results. Although the number of exclusions is small, the fact that the reasons for exclusion may be potentially related to the study outcomes is particularly troublesome, as it may signal the occurrence of strong bias.

## Conclusion

For inducing spinal anesthesia for caesarean section, combined use of hyperbaric (4 mg) and hypobaric (6 mg) ropivacaine achieved satisfactory anesthesia, longer induction time, a lower level of sensory blockade, and fewer incidences of hypotension, nausea, or vomiting relative to single hyperbaric ropivacaine.

## Supporting Information

S1 CONSORT Checklist CONSORT Checklist. (DOC)

S1 Protocol Trial Protocol. (DOC)

S1 Protocol Translation Protocol Translation. (DOC)
