# Correction: Combined Use of Hyperbaric and Hypobaric Ropivacaine Significantly Improves Hemodynamic Characteristics in Spinal Anesthesia for Caesarean Section: A Prospective, Double-Blind, Randomized, Controlled Study

**DOI:** 10.1371/journal.pone.0132082

**Published:** 2015-06-25

**Authors:** The PLOS ONE Staff

The affiliation for the first two authors is incorrect. ZheFeng Quan and Ming Tian are not affiliated with #1 but with #2 Department of Anesthesia, Beijing Friendship Hospital, Capital Medical University, Beijing, 100050, China. The publisher apologizes for the error.

## References

[1] Quan Z, Tian M, Chi P, Li X, He H, Luo C (2015) Combined Use of Hyperbaric and Hypobaric Ropivacaine Significantly Improves Hemodynamic Characteristics in Spinal Anesthesia for Caesarean Section: A Prospective, Double-Blind, Randomized, Controlled Study. PLoS ONE 10(5): e0125014 doi: 10.1371/journal.pone.0125014 25970485 10.1371/journal.pone.0125014 PMC4430289

