# Supplementary material for: Combined Use of Hyperbaric and Hypobaric Ropivacaine Significantly Improves Hemodynamic Characteristics in Spinal Anesthesia for Caesarean Section: A Prospective, Double-Blind, Randomized, Controlled Study
Source: PLoS One. 2015 May 13;10(5):e0125014. doi: 10.1371/journal.pone.0125014 (PMC4430289; doi:10.1371/journal.pone.0125014)
Supplement: S1 Protocol — (DOC) [file pone.0125014.s002.doc]

**首都医科大学附属北京佑安医院**

**伦理申请书**

# **课题名称：**联合使用重比重和轻比重罗哌卡因用于剖宫产腰麻手术能显著改善产妇的血流动力学变化

# **申 请 人： 权哲峰**

**所在科室：麻醉科**

**联系电话： 010-83997140**

**电子信箱： shimane126@126.com**

**申报日期：2013年4月5日**

**起止年限：2013年～2014年**

**首都医科大学附属北京佑安医院**

**二〇一三年四月制**

**一、基本信息**

| **申**  **请**  **人**  **信**  **息** | **姓名** | 权哲峰 | **性别** | **男** | **出生年月** | 1976年04月23日 |
| --- | --- | --- | --- | --- | --- | --- |
|  | **学位** | 硕士 | **职称** | **主治医师** | **主要研究领域** | 产科麻醉 |
| **课题基本信息** | **项目名称** | **中 文** | 联合使用重比重和轻比重罗哌卡因用于剖宫产腰麻手术能显著改善产妇的血流动力学变化 | | | |
|  |  | **英 文** | Combined use of hyperbaric and hypobaric ropivacaine significantly improves hemodynamic characteristics in spinal anesthesia for caesarean section | | | |
|  | **中文关键词** | 腰麻，重比重罗哌卡因，轻比重罗哌卡因，剖宫产术 | | | **英文关键词** | spinal anesthesia, hyperbaric ropivacaine, hypobaric ropivacaine, caesarean section |

**二、课题内容简介（400字以内）**

| 本研究探索一种降低腰麻剖宫产术中低血压发生率的新技术，从而降低胎盘供血不足导致胎儿窘迫及新生儿死亡发生率的增加（特别是降低患有妊娠期高血压疾病产妇的新生儿死亡率）。腰麻最大缺点是上界阻滞平面不易控制，进而低血压发生率高。这主要原因是我们无法定量控制局麻药物向头侧扩散的剂量，进而无法改变上界阻滞平面不易控制的情况，增加了低血压发生率。  本研究的创新点在于腰麻药物的配制新技术，既采用了重比重联合轻比重的独特方法。这种新技术利用人体的正常生理曲线达到定量控制药物去向的目的。其机制是既先注入的重比重药液会向头侧扩散（决定麻醉上界阻滞平面），而之后给予的轻比重会向尾侧扩散（决定麻醉下界阻滞平面）。这样我们就可以利用重比重的药物剂量来达到控制麻醉上界阻滞平面的作用，从而解决腰麻上界感觉阻滞平面不易控制，降低低血压发生率的情况，从而改善胎盘供血不足导致胎儿窘迫及新生儿死亡发生率的增高。 |
| --- |

**三、课题组人员基本情况（不包括申请人）**

| **序号** | **姓名** | **出生**  **年月** | **性 别** | **职称/职务** | **学位** | **所在科室** | **任务分工** | **签字** |
| --- | --- | --- | --- | --- | --- | --- | --- | --- |
| 1 | 李昕 |  | 男 | 副主任医师 | 本科 | 麻醉科 | 实 施麻醉 |  |
| **2** | 贺海丽 |  | 女 | 医师 | 本科 | 麻醉科 | 实施麻醉 |  |
| **3** | 彭科军 |  | 男 | 主治医师 | 硕士 | 麻醉科 | 实施麻醉兼统计分析 |  |
| **4** | 彭健 |  | 女 | 护师 | 大专 | 麻醉科 | 配置药品兼收集数据 |  |
| 5 | 池萍 |  | 女 | 主任医师 | 博士 | 麻醉科 | 实验管理 |  |
| 6 | 田鸣 |  | 男 | 主任医师 | 博士 | 麻醉科 | 实验设计 |  |

## **四、立题依据（**主要描述研究意义，包括研究涉及的相关健康问题是否严重？为什么该问题会长期存在？目前该领域现有的知识技术水平？该研究项目是否能产生出比现有技术手段更好的干预措施？国内外研究现状及发展动态分析，请附近五年主要参考文献）

| 椎管内麻醉无疑是择期剖宫产最佳的麻醉方式。因为与全麻相比可降低误吸、插管失败风险及产妇死亡率。而椎管内麻醉又可分为硬膜外麻醉和腰麻。其中因腰麻起效迅速、镇痛效果确切而成为择期剖宫产的主流麻醉方式，而腰麻的最大缺点是上界阻滞平面不易控制，低血压发生率高。而低血压通过降低胎盘供血不足导致胎儿窘迫及新生儿死亡发生率的增加，特别是增加患有妊娠期高血压疾病产妇的新生儿死亡率（可高达15%～30%）。因此，如何降低产妇的低血压发生率是一个迫切需要解决的现实问题，也是目前产科麻醉领域最大的研究热点。  这种方法简单、易行，又避免了像血管活性药物一样的不良反应，而且使用成本低廉易于普及，将成为腰麻剖宫产术中单独的一种麻醉方式既联合式腰麻。  这种方法其最大的优点在于将不利的人体生理曲线变成了有利的因素，以定量、定向的方式控制局麻药物的分布，从而改善腰麻不易控制麻醉上界阻滞平面的情况，降低低血压的发生率，进而降低胎盘供血不足导致胎儿窘迫及新生儿死亡发生率的增加。这种环保低成本的麻醉方法将成腰麻剖宫产中的主流麻醉方式。  **部分参考文献**  [1]. Sng BL, Tan HS, Sia AT.Closed-loop double-vasopressor automated system vs manual bolus vasopressor to treat hypotension during spinal anaesthesia for caesarean section: a randomised controlled trial.Anaesthesia. 2014 Jan;69(1):37-45.  [2]. Dalchow S, Lubeigt O, Peters G, Harvey A, Duggan T, Binning A.Transcutaneous carbon dioxide levels and oxygen saturation following caesarean section performed under spinal anaesthesia with intrathecal opioids. Int J Obstet Anesth. 2013 Jul;22(3):217-22.  [3]. Toyama S, Kakumoto M, Morioka M, Matsuoka K, Omatsu H, Tagaito Y, Numai T, Shimoyama M.Perfusion index derived from a pulse oximeter can predict the incidence of hypotension during spinal anaesthesia for Caesarean delivery.Br J Anaesth. 2013 Aug;111(2):235-41.  [4]. Cardoso MM, Leite AO, Santos EA, Gozzani JL, Mathias LA.Effect of dexamethasone on prevention of postoperative nausea, vomiting and pain after caesarean section: a randomised, placebo-controlled, double-blind trial.Eur J Anaesthesiol. 2013 Mar;30(3):102-5.  [5]. Kitahara T, Kuri S, Yoshida J. The spread of drugs used for spinal anesthesia. Anesthesiology 1956;17:205–8  [6]. Carpenter RL, Hogan QH, Liu SS, Crane B, Moore J. Lumbosacral cerebrospinal fluid volume is the primary determinant of sensory block extent and duration during spinal anesthesia. Anesthesiology 1998;89:24–9  [7]. Higuchi H, Hirata J, Adachi Y, Kazama T. Influence of lumbosacral cerebrospinal fluid density, velocity, and volume on extent and duration of plain bupivacaine spinal anesthesia. Anesthesiology 2004;100:106–14  [8]. Horlocker TT, Wedel DJ. Density, specific gravity, and baricity of spinal anesthetic solutions at body temperature. Anesth Analg 1993;76:1015–8  [9]. Heller AR, Zimmermann K, Seele K, Rossel T, Koch T, Litz RJ. Modifying the baricity of local anesthetics for spinal anesthesia by temperature adjustment: model calculations. Anesthesiology 2006;105:346–53  [10]. Lumbosacral cerebrospinal fluid volume in Humans using three-dimentional magnetic resonance imaging Anaesthesia & analgesia 2006;103(5):1306~1310.  [11] Apaydin Y, Erk G, Sacan O, Tiryaki C, Taspinar V.Characteristics of unilateral spinal anesthesia at different speeds of intrathecal injection.J Anesth. 2011 Jun;25(3):380-5.  [12] Kaya M, Oztürk I, Tuncel G, Senel GO, Eski?irak H, Kadio?ullari N.A comparison of low dose hyperbaric levobupivacaine and hypobaric levobupivacaine in unilateral spinal anaesthesia.Anaesth Intensive Care. 2010 Nov;38(6):1002-7.  [13].Rosseland LA, Hauge TH, Grindheim G, Stubhaug A, Langes?ter E.Changes in blood pressure and cardiac output during cesarean delivery: the effects of oxytocin and carbetocin compared with placebo.Anesthesiology. 2013 Sep;119(3):541-51.  [14].Toyama S, Kakumoto M, Morioka M, Matsuoka K, Omatsu H, Tagaito Y, Numai T, Shimoyama M.Perfusion index derived from a pulse oximeter can predict the incidence of hypotension during spinal anaesthesia for Caesarean delivery.Br J Anaesth. 2013 Aug;111(2):235-41.  [15].Bhardwaj N, Jain K, Arora S, Bharti N.A comparison of three vasopressors for tight control of maternal blood pressure during cesarean section under spinal anesthesia: Effect on maternal and fetal outcome.J Anaesthesiol Clin Pharmacol. 2013 Jan;29(1):26-31.  [16].Sia AT, Tan HS, Sng BL.Closed-loop double-vasopressor automated system to treat hypotension during spinal anaesthesia for caesarean section: a preliminary study.Anaesthesia. 2012 Dec;67(12):1348-55.  [17].Van de Velde M, Van Schoubroeck D, Jani J, Teunkens A, Missant C, Deprest J.Combined spinal-epidural anesthesia for cesarean delivery: dose-dependent effects of hyperbaric bupivacaine on maternal hemodynamics.Anesth Analg. 2006 Jul;103(1):187-90.  [18]. Teoh WH, Thomas E, Tan HM.Ultra-low dose combined spinal-epidural anesthesia with intrathecal bupivacaine 3.75 mg for cesarean delivery: a randomized controlled trial.Int J Obstet Anesth. 2006 Oct;15(4):273-8.  [19].El-HakeemEE, KakiAM, AlmazrooaAA, Al-MansouriNM, AlhashemiJA.Effects of sitting up for five minutes versus immediately lying down after spinal anesthesia for Cesarean deliveryon fluid and ephedrine requirement; a randomized trial. Can J Anaesth. 2011 Dec;58(12):1083-9.  [20]. Loubert C, Hallworth S, Fernando R, Columb M, Patel N, Sarang K, Sodhi V. Does the baricity of bupivacaine influence intrathecal spread in the prolonged sitting position before electivecesarean delivery? A prospective randomized controlled study. Anesth Analg . 2011 Oct; 113 (4) :811-7.  [21] Obasuyi BI, Fyneface-Ogan S, Mato CN.A comparison of the haemodynamic effects of lateral and sitting positions during induction of spinal anaesthesiafor caesarean section Int J Obstet Anesth 2013 Apr; 22 (2) :124-8 .  [22].Akhtar MN, Tariq S, Abbas N, Murtaza G, Nadeem Naqvi SM.Comparison of haemodynamic changes in patients undergoing unilateral and bilateral spinal anaesthesia.J Coll Physicians Surg Pak. 2012 Dec;22(12):747-50.  [23].Obasuyi BI, Fyneface-Ogan S, Mato CN.A comparison of the haemodynamic effects of lateral and sitting positions during induction of spinal anaesthesia for caesarean section.Int J Obstet Anesth. 2013 Apr;22(2):124-8.  [24].Kim JT, Lee JH, Cho CW, Kim HC, Bahk JH.The influence of spinal flexion in the lateral decubitus position on the unilaterality of spinal anesthesia. Anesth Analg. 2013 Oct;117(4):1017-21.  [25].Atef H, El-Kasaby Ael-D, Omera M, Badr M.Optimal dose of hyperbaric bupivacaine 0.5% for unilateral spinal anesthesia during diagnostic knee arthroscopy.Middle East J Anesthesiol. 2012 Feb;21(4):591-8.  [26].Kim JT, Lee JH, Cho CW, Kim HC, Bahk JH. The influence of spinal flexion in the lateral decubitus position on the unilaterality of spinal anesthesia. Anesth Analg. 2013 Oct;117(4):1017-21.  [27].Lilot M, Meuret P, Bouvet L, Caruso L, Dabouz R, Deléat-Besson R, Rousselet B, Thouverez B, Zadam A, Allaouchiche B, Boselli E. Hypobaric spinal anesthesia with ropivacaine plus sufentanil for traumatic femoral neck surgery in the elderly: a dose-response study. Anesth Analg. 2013 Jul;117(1):259-64.  [28].Apaydin Y, Erk G, Sacan O, Tiryaki C, Taspinar V.Characteristics of unilateral spinal anesthesia at different speeds of intrathecal injection.J Anesth. 2011 Jun;25(3):380-5.  [29].G.LeoS, SngBL, LimY, SiaAT.A randomized comparison of low doses of hyperbaric bupivacaine in combined spinal-epidural anesthesia forcesarean delivery. Anesth Analg. 2009 Nov;109(5):1600-5.  [30]..Maayan-Metzger A, Schushan-Eisen I, Todris L, Etchin A, Kuint J. Maternal hypotension during elective cesarean section and short-term neonatal outcome. Am J Obstet Gynecol 2010; 202: 5-56.  [31]..El-HakeemEE, KakiAM, AlmazrooaAA, Al-MansouriNM, AlhashemiJA.Effects of sitting up for five minutes versus immediately lying down after spinal anesthesia for Cesarean deliveryon fluid and ephedrine requirement; a randomized trial. Can J Anaesth. 2011 Dec;58(12):1083-9.  [32]. Coppejans HC1, Hendrickx E, Goossens J, Vercauteren MP. The sitting versus right lateral position during combined spinal-epidural anesthesia for cesarean delivery: blockcharacteristics and severity of hypotension. Anesth Analg. 2006 Jan;102(1):243-7.  [33].Rosseland LA1, Hauge TH, Grindheim G, Stubhaug A, Langes?ter E. Changes in blood pressure and cardiac output during cesarean delivery: the effects of oxytocin and carbetocincompared with placebo. Anesthesiology. 2013 Sep;119(3):541-51. |
| --- |

**五、研究目标**

| **（一）总体目标** 降低产妇低血压发生率。 |
| --- |

**六、课题设计**

| **（一）研究内容与方法（此部分为重点阐述内容，包括有关研究方法、实验手段、关键技术、临床研究的研究对象、样本量、入排标准、观察指标和时点等）**    方法：  选择140例拟腰硬联合麻醉下择期剖宫产的ASA分级 I级和Ⅱ级的足月单胎（孕周≥37周）妊娠妇女。  排除包括：年龄18岁以下，身高<150cm 或> 180cm，体重<50Kg或> 100Kg，高血压、多胎妊娠、前置胎盘、心脑血管疾病、已知胎儿发育异常、椎管内麻醉禁忌证或有产程开始征象的患者。  根据计算机编码将患者随机分为联合组（A组）和对照组（B组），有一名不参与麻醉实施及观察的研究者根据随机结果分配患者进行分组，并负责配制所需要的腰麻药物。所有患者腰麻药物均分成两个注射器，分别命名为1号注射器和2号注射器，具体配制如下；  A组  1号注射器：0.5% ropivacaine, 0.8 mL (其中含fentanyl 4ug and 4% glucose)  2号注射器：0.5% ropivacaine, 1.2 mL（其中含fentanyl 6ug and sterile distilled water）  B组  1号注射器：0.5% ropivacaine, 0.8ml （其中含fentanyl 4ug and 4% glucose）  2号注射器：0.5% ropivacaine, 1.2 mL（其中含fentanyl 6ug and sterile distilled water）  所以孕妇采取右侧卧并在后背消毒后，选择在L2-3 间隙行腰-硬联合穿刺，在确认腰麻穿刺针进入蛛网膜下腔后先后注射1号（0.8ml）、2号（1.2ml）注射器里的药物,向蛛网膜下腔注射的速度为0.1ml/S。退出腰麻针向硬膜外腔置入硬膜外导管3cm，固定导管，  记录两组患者的血流动力学变化、感觉阻滞的水平、硬膜外导管置入时间、完全镇痛时间（从鞘内注射的时间开始到VAS评分>0的时间）、麻黄碱平均使用剂量、麻醉诱导时间(从腰麻药物注射完毕开始至感觉阻滞平面达到T8时)手术时间、出血量、尿量及各种不良反应（包括恶心，呕吐，发抖，头晕）。同时记录新生儿1分钟和5分钟时的Apgar评分及脐带A、V血气。  选择如下技术路线： |
| --- |
| **（二）技术路线**  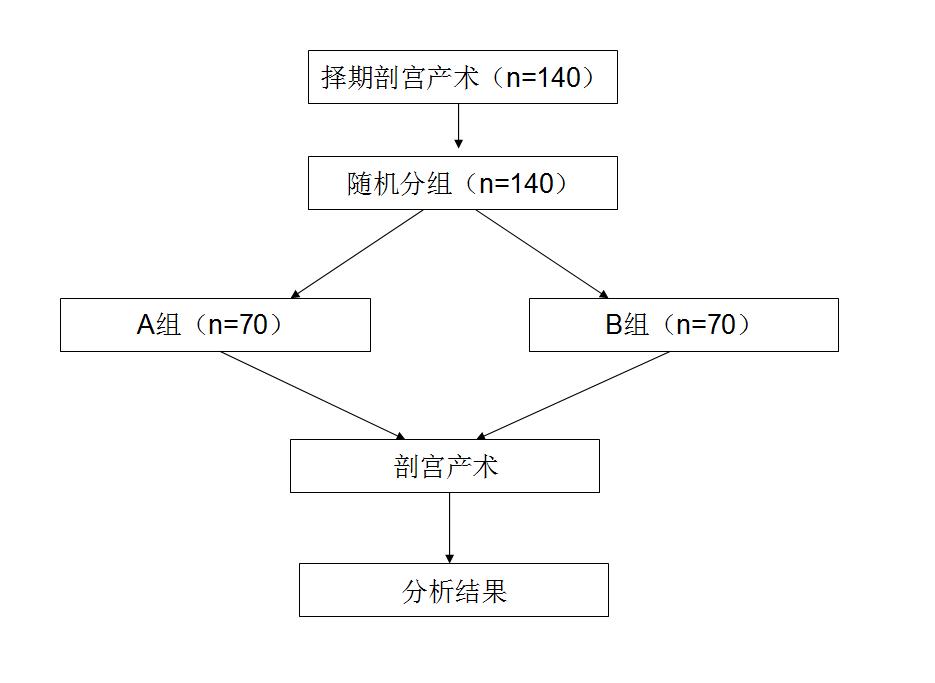 |
| **（三）研究难点与对策**    根据随机表严格按照随机顺序进行入组 |

**七、研究伦理**（涉及人类受试者的研究需填写）

| 1. 临床常用药物  2. 知情同意 |
| --- |

**八、特色与创新**（包括课题的研究目标产出、研究方法、研究技术等方面的创新点）

| 腰麻的最大缺点是麻醉上界阻滞平面不易控制，低血压发生率高。导致围术期新生儿发生酸中毒及胎儿死亡率增加。  虽然目前通过改变腰椎穿刺间隙，调整腰麻针针孔方向，控制局麻药物的注射速度及改变患者的体位的方法，但依然无法定量控制局麻药物向头侧扩散的剂量，从而无法改变上界阻滞平面不易控制的情况。  本研究探索一种腰麻剖宫产术中的全新技术，是一种联合不同比重腰麻药物（重比重联合轻比重）的方法。首先，我们在剖宫产术中选择L2～3椎间隙进行穿刺，先给与重比重再给与轻比重。当患者平躺时重比重和轻比重分别向头侧和尾侧移动，从而达到了控制局麻药物定向、定量的目的，有利于控制局麻药物向头侧扩散的剂量，有利于上界阻滞平面的控制，降低低血压发生率，从而改善胎盘供血不足导致胎儿窘迫及新生儿死亡发生率的增高。 |
| --- |
