# Supplementary material for: Combined Use of Hyperbaric and Hypobaric Ropivacaine Significantly Improves Hemodynamic Characteristics in Spinal Anesthesia for Caesarean Section: A Prospective, Double-Blind, Randomized, Controlled Study
Source: PLoS One. 2015 May 13;10(5):e0125014. doi: 10.1371/journal.pone.0125014 (PMC4430289; doi:10.1371/journal.pone.0125014)
Supplement: S1 Protocol Translation — (DOC) [file pone.0125014.s003.doc]

**BEIJING YOU N HOSPITAL CAPITAL MEDICAL UNIVERSITY**

**Title：**Combined use of hyperbaric and hypobaric ropivacaine significantly improves hemodynamic characteristics in spinal anesthesia for caesarean section: A prospective, double-blind, randomized, controlled study

# **Applicant：**Zhe-Feng Quan

**Department：**Department of Anesthesiology

**TEL：** 010-83997140

**E-mail：** shimane126@126.com

**Reporting date：**Apr,15,2013

**Starting and ending time：**2013～2014

Beijing YouAn Hospital, Capital Medical University

**2013 Years April**

**一、The basic information**

| **申**  **请**  **人**  **信**  **息** | **name** | Zhefeng quan | **性别** | **男** | **出生年月** | 1976年04月23日 |
| --- | --- | --- | --- | --- | --- | --- |
|  | **学位** | 硕士 | **职称** | **主治医师** | **主要研究领域** | 产科麻醉 |
| **课题基本信息** | **项目名称** | **中 文** | 联合使用重比重和轻比重罗哌卡因用于剖宫产腰麻手术能显著改善产妇的血流动力学变化 | | | |
|  |  | **英 文** | Combined use of hyperbaric and hypobaric ropivacaine significantly improves hemodynamic characteristics in spinal anesthesia for caesarean section | | | |
|  | **中文关键词** | 腰麻，重比重罗哌卡因，轻比重罗哌卡因，剖宫产术 | | | **英文关键词** | spinal anesthesia, hyperbaric ropivacaine, hypobaric ropivacaine, caesarean section |

二、Project brief introduction（400字以内）

| We hypothesized that the unwanted effect of hypotension associated with use of hyperbaric anesthetics for spinal anesthesia during caesarean section may be ameliorated by utilizing the properties of both hyperbaric and hypobaric solutions, specifically by administering 4 mg of hyperbaric anesthetic and 6 mg of hypobaric anesthetic successively into the intrathecal space through the L2-3 interspace. Theoretically, the spread of the first 4 mg of hyperbaric anesthetic would move in the cephalad direction (subsiding towards T6), and the 6 mg of hypobaric anesthetic would be inclined to spread caudally (floating towards L3). This unique technique could avoid the spinal characteristic moving to one side and easily control the upper plane of anesthesia by adjusting the dose of hypobaric anesthetics. |
| --- |

**三、Other researchers**

| **序号** | **姓名** | **出生**  **年月** | **性 别** | **职称/职务** | **学位** | **所在科室** | **任务分工** | **签字** |
| --- | --- | --- | --- | --- | --- | --- | --- | --- |
| 1 | 李昕 |  | 男 | 副主任医师 | 本科 | 麻醉科 | 实 施麻醉 |  |
| **2** | 贺海丽 |  | 女 | 医师 | 本科 | 麻醉科 | 实施麻醉 |  |
| **3** | 彭科军 |  | 男 | 主治医师 | 硕士 | 麻醉科 | 实施麻醉兼统计分析 |  |
| **4** | 彭健 |  | 女 | 护师 | 大专 | 麻醉科 | 配置药品兼收集数据 |  |
| 5 | 池萍 |  | 女 | 主任医师 | 博士 | 麻醉科 | 实验管理 |  |
| 6 | 田鸣 |  | 男 | 主任医师 | 博士 | 麻醉科 | 实验设计 |  |

## **四、Project basis**

| Spinal anesthesia is currently more utilized in patients undergoing caesarean section1-4. However, uncontrollability of upper plane of anesthesia and higher incidence of hypotension are the main drawbacks during anesthesia, which are associated with the natural physiological curve of the human body and the inability of anesthesiologists to accurately identify lumbar interspaces. During spinal anesthesia for caesarean section, it is difficult to ascertain an accurate injection site which is in L2-3 or L3-4 interspace.  We hypothesized that the unwanted effect of hypotension associated with use of hyperbaric anesthetics for spinal anesthesia during caesarean section may be ameliorated by utilizing the properties of both hyperbaric and hypobaric solutions, specifically by administering 4 mg of hyperbaric anesthetic and 6 mg of hypobaric anesthetic successively into the intrathecal space through the L2-3 interspace. Theoretically, the spread of the first 4 mg of hyperbaric anesthetic would move in the cephalad direction (subsiding towards T6), and the 6 mg of hypobaric anesthetic would be inclined to spread caudally (floating towards L3). This unique technique could avoid the spinal characteristic moving to one side and easily control the upper plane of anesthesia by adjusting the dose of hypobaric anesthetics.  Moreover, this unique technique, unlike isobaric anesthetics, would not be affected by the physical properties of cerebrospinal fluid5-9, which could lead to an unpredictability of the level of sensory blockade10. Therefore, the purpose of the present study was to compare the anesthetic effects and hemodynamic characteristics of the combined use of hyperbaric and hypobaric ropivacaine with a single hyperbaric ropivacaine administration during spinal anesthesia for caesarean section.  **References**  [1]. Sng BL, Tan HS, Sia AT.Closed-loop double-vasopressor automated system vs manual bolus vasopressor to treat hypotension during spinal anaesthesia for caesarean section: a randomised controlled trial.Anaesthesia. 2014 Jan;69(1):37-45.  [2]. Dalchow S, Lubeigt O, Peters G, Harvey A, Duggan T, Binning A.Transcutaneous carbon dioxide levels and oxygen saturation following caesarean section performed under spinal anaesthesia with intrathecal opioids. Int J Obstet Anesth. 2013 Jul;22(3):217-22.  [3]. Toyama S, Kakumoto M, Morioka M, Matsuoka K, Omatsu H, Tagaito Y, Numai T, Shimoyama M.Perfusion index derived from a pulse oximeter can predict the incidence of hypotension during spinal anaesthesia for Caesarean delivery.Br J Anaesth. 2013 Aug;111(2):235-41.  [4]. Cardoso MM, Leite AO, Santos EA, Gozzani JL, Mathias LA.Effect of dexamethasone on prevention of postoperative nausea, vomiting and pain after caesarean section: a randomised, placebo-controlled, double-blind trial.Eur J Anaesthesiol. 2013 Mar;30(3):102-5.  [5]. Kitahara T, Kuri S, Yoshida J. The spread of drugs used for spinal anesthesia. Anesthesiology 1956;17:205–8  [6]. Carpenter RL, Hogan QH, Liu SS, Crane B, Moore J. Lumbosacral cerebrospinal fluid volume is the primary determinant of sensory block extent and duration during spinal anesthesia. Anesthesiology 1998;89:24–9  [7]. Higuchi H, Hirata J, Adachi Y, Kazama T. Influence of lumbosacral cerebrospinal fluid density, velocity, and volume on extent and duration of plain bupivacaine spinal anesthesia. Anesthesiology 2004;100:106–14  [8]. Horlocker TT, Wedel DJ. Density, specific gravity, and baricity of spinal anesthetic solutions at body temperature. Anesth Analg 1993;76:1015–8  [9]. Heller AR, Zimmermann K, Seele K, Rossel T, Koch T, Litz RJ. Modifying the baricity of local anesthetics for spinal anesthesia by temperature adjustment: model calculations. Anesthesiology 2006;105:346–53  [10]. Lumbosacral cerebrospinal fluid volume in Humans using three-dimentional magnetic resonance imaging Anaesthesia & analgesia 2006;103(5):1306~1310.  [11] Apaydin Y, Erk G, Sacan O, Tiryaki C, Taspinar V.Characteristics of unilateral spinal anesthesia at different speeds of intrathecal injection.J Anesth. 2011 Jun;25(3):380-5.  [12] Kaya M, Oztürk I, Tuncel G, Senel GO, Eski?irak H, Kadio?ullari N.A comparison of low dose hyperbaric levobupivacaine and hypobaric levobupivacaine in unilateral spinal anaesthesia.Anaesth Intensive Care. 2010 Nov;38(6):1002-7.  [13].Rosseland LA, Hauge TH, Grindheim G, Stubhaug A, Langes?ter E.Changes in blood pressure and cardiac output during cesarean delivery: the effects of oxytocin and carbetocin compared with placebo.Anesthesiology. 2013 Sep;119(3):541-51.  [14].Toyama S, Kakumoto M, Morioka M, Matsuoka K, Omatsu H, Tagaito Y, Numai T, Shimoyama M.Perfusion index derived from a pulse oximeter can predict the incidence of hypotension during spinal anaesthesia for Caesarean delivery.Br J Anaesth. 2013 Aug;111(2):235-41.  [15].Bhardwaj N, Jain K, Arora S, Bharti N.A comparison of three vasopressors for tight control of maternal blood pressure during cesarean section under spinal anesthesia: Effect on maternal and fetal outcome.J Anaesthesiol Clin Pharmacol. 2013 Jan;29(1):26-31.  [16].Sia AT, Tan HS, Sng BL.Closed-loop double-vasopressor automated system to treat hypotension during spinal anaesthesia for caesarean section: a preliminary study.Anaesthesia. 2012 Dec;67(12):1348-55.  [17].Van de Velde M, Van Schoubroeck D, Jani J, Teunkens A, Missant C, Deprest J.Combined spinal-epidural anesthesia for cesarean delivery: dose-dependent effects of hyperbaric bupivacaine on maternal hemodynamics.Anesth Analg. 2006 Jul;103(1):187-90.  [18]. Teoh WH, Thomas E, Tan HM.Ultra-low dose combined spinal-epidural anesthesia with intrathecal bupivacaine 3.75 mg for cesarean delivery: a randomized controlled trial.Int J Obstet Anesth. 2006 Oct;15(4):273-8.  [19].El-HakeemEE, KakiAM, AlmazrooaAA, Al-MansouriNM, AlhashemiJA.Effects of sitting up for five minutes versus immediately lying down after spinal anesthesia for Cesarean deliveryon fluid and ephedrine requirement; a randomized trial. Can J Anaesth. 2011 Dec;58(12):1083-9.  [20]. Loubert C, Hallworth S, Fernando R, Columb M, Patel N, Sarang K, Sodhi V. Does the baricity of bupivacaine influence intrathecal spread in the prolonged sitting position before electivecesarean delivery? A prospective randomized controlled study. Anesth Analg . 2011 Oct; 113 (4) :811-7.  [21] Obasuyi BI, Fyneface-Ogan S, Mato CN.A comparison of the haemodynamic effects of lateral and sitting positions during induction of spinal anaesthesiafor caesarean section Int J Obstet Anesth 2013 Apr; 22 (2) :124-8 .  [22].Akhtar MN, Tariq S, Abbas N, Murtaza G, Nadeem Naqvi SM.Comparison of haemodynamic changes in patients undergoing unilateral and bilateral spinal anaesthesia.J Coll Physicians Surg Pak. 2012 Dec;22(12):747-50.  [23].Obasuyi BI, Fyneface-Ogan S, Mato CN.A comparison of the haemodynamic effects of lateral and sitting positions during induction of spinal anaesthesia for caesarean section.Int J Obstet Anesth. 2013 Apr;22(2):124-8.  [24].Kim JT, Lee JH, Cho CW, Kim HC, Bahk JH.The influence of spinal flexion in the lateral decubitus position on the unilaterality of spinal anesthesia. Anesth Analg. 2013 Oct;117(4):1017-21.  [25].Atef H, El-Kasaby Ael-D, Omera M, Badr M.Optimal dose of hyperbaric bupivacaine 0.5% for unilateral spinal anesthesia during diagnostic knee arthroscopy.Middle East J Anesthesiol. 2012 Feb;21(4):591-8.  [26].Kim JT, Lee JH, Cho CW, Kim HC, Bahk JH. The influence of spinal flexion in the lateral decubitus position on the unilaterality of spinal anesthesia. Anesth Analg. 2013 Oct;117(4):1017-21.  [27].Lilot M, Meuret P, Bouvet L, Caruso L, Dabouz R, Deléat-Besson R, Rousselet B, Thouverez B, Zadam A, Allaouchiche B, Boselli E. Hypobaric spinal anesthesia with ropivacaine plus sufentanil for traumatic femoral neck surgery in the elderly: a dose-response study. Anesth Analg. 2013 Jul;117(1):259-64.  [28].Apaydin Y, Erk G, Sacan O, Tiryaki C, Taspinar V.Characteristics of unilateral spinal anesthesia at different speeds of intrathecal injection.J Anesth. 2011 Jun;25(3):380-5.  [29].G.LeoS, SngBL, LimY, SiaAT.A randomized comparison of low doses of hyperbaric bupivacaine in combined spinal-epidural anesthesia forcesarean delivery. Anesth Analg. 2009 Nov;109(5):1600-5.  [30]..Maayan-Metzger A, Schushan-Eisen I, Todris L, Etchin A, Kuint J. Maternal hypotension during elective cesarean section and short-term neonatal outcome. Am J Obstet Gynecol 2010; 202: 5-56.  [31]..El-HakeemEE, KakiAM, AlmazrooaAA, Al-MansouriNM, AlhashemiJA.Effects of sitting up for five minutes versus immediately lying down after spinal anesthesia for Cesarean deliveryon fluid and ephedrine requirement; a randomized trial. Can J Anaesth. 2011 Dec;58(12):1083-9.  [32]. Coppejans HC1, Hendrickx E, Goossens J, Vercauteren MP. The sitting versus right lateral position during combined spinal-epidural anesthesia for cesarean delivery: blockcharacteristics and severity of hypotension. Anesth Analg. 2006 Jan;102(1):243-7.  [33].Rosseland LA1, Hauge TH, Grindheim G, Stubhaug A, Langes?ter E. Changes in blood pressure and cardiac output during cesarean delivery: the effects of oxytocin and carbetocincompared with placebo. Anesthesiology. 2013 Sep;119(3):541-51. |
| --- |

**五、Research objectives**

| Reduce the incidence of hypotension |
| --- |

**六、Study design**

| Methods    this study were 140 patients, ASA physical status I or II (gestational age ≥37 weeks, singleton) undergoing elective cesarean delivery under combined spinal-epidural anesthesia.  The exclusion criteria were: age <18 years; height <150 cm or >180 cm; weight <50 kg or >100 kg; hypertension; multiple pregnancy; placenta previa; cardiovascular or cerebrovascular diseases; known abnormal fetal development; contraindications for intraspinal anesthesia; or signs of labor onset.  Patients were randomly allocated into two groups (Group A: Combined use of hyperbaric and hypobaric ropivacaine; Group B: Controls) using a computer-generated table. The investigator responsible for the random allocation and preparation of spinal anesthetics was not present during surgery or postoperative evaluations.  The anesthetics used in both groups consisted of two parts. Group A received from syringe A1, 0.8 mL of 0.5% hyperbaric ropivacaine, containing 4 μg fentanyl and 4% glucose; and from syringe A2, 1.2 mL of 0.5% hypobaric ropivacaine, containing 6 μg fentanyl and sterile distilled water. Group B, the control, received from syringe B1, 0.8 mL of 0.5% hyperbaric ropivacaine, containing 4 μg fentanyl and 4% glucose; and from syringe B2, 1.2 mL of 0.5% hypobaric ropivacaine, containing 6 μg fentanyl and 4% glucose.  A combined spinal-epidural procedure was performed at the L2-3 interspace with the patient maintained in the right lateral decubitus position. In each group of patients, the anesthetics in syringes 1 and 2 were injected successively when the needle entered the subarachnoid space. Injection speed in both groups was 0.1 mL/s. The spinal anesthesia needle was withdrawn and 3 cm of the epidural catheter was placed into the epidural space.  recorded: hemodynamic data, upper sensory blockade level, insertion time of the epidural catheter, complete analgesia time [beginning of intrathecal injection to a visual analogue scale (VAS) >0], mean dose of ephedrine, time from the end of intrathecal injection to achievement of T8 sensory blockade level, operative time, volume of intraoperative blood loss, volume of intraoperative urine, and incidence of complications (nausea, vomiting, shivering, or dizziness). Neonatal conditions were assessed using Apgar scores at 1 and 5 min, and blood gas analyses of the umbilical artery and vein were also performed. |
| --- |
| **（二）**Technology Roadmap  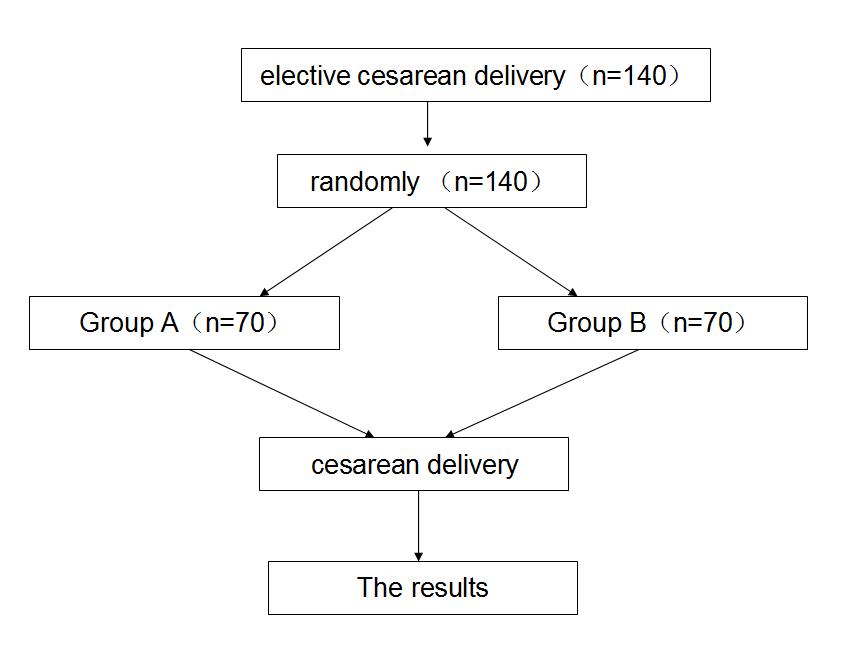 |
| **（三）Research Difficulties**    Randomization |

**七、Research Ethics**

| 1. Clinical commonly used drugs  2. Informed Consent |
| --- |

**八、Innovation**

| No reports have shown the effects of combined use of hyperbaric and hypobaric solutions in spinal anesthesia. The purpose of the present study was to compare the anesthetic effects and hemodynamic characteristics in the combined use of hyperbaric and hypobaric ropivacaine with single hyperbaric ropivacaine for caesarean section. |
| --- |
